# Novel approaches for periodontal tissue engineering

**DOI:** 10.1002/dvg.23499

**Published:** 2022-09-10

**Authors:** W. Benton Swanson, Yao Yao, Yuji Mishina

**Affiliations:** ^1^ Department of Biologic and Materials Science, Division of Prosthodontics University of Michigan School of Dentistry Ann Arbor Michigan USA; ^2^ Department of Periodontics and Oral Medicine University of Michigan School of Dentistry Ann Arbor Michigan USA; ^3^ Biointerfaces Institute University of Michigan Ann Arbor Michigan USA

**Keywords:** neural crest, periodontology, teeth, tissue, tissue engineering

## Abstract

The periodontal complex involves the hard and soft tissues which support dentition, comprised of cementum, bone, and the periodontal ligament (PDL). Periodontitis, a prevalent infectious disease of the periodontium, threatens the integrity of these tissues and causes irreversible damage. Periodontal therapy aims to repair and ultimately regenerate these tissues toward preserving native dentition and improving the physiologic integration of dental implants. The PDL contains multipotent stem cells, which have a robust capacity to differentiate into various types of cells to form the PDL, cementum, and alveolar bone. Selection of appropriate growth factors and biomaterial matrices to facilitate periodontal regeneration are critical to recapitulate the physiologic organization and function of the periodontal complex. Herein, we discuss the current state of clinical periodontal regeneration including a review of FDA‐approved growth factors. We will highlight advances in preclinical research toward identifying additional growth factors capable of robust repair and biomaterial matrices to augment regeneration similarly and synergistically, ultimately improving periodontal regeneration's predictability and long‐term efficacy. This review should improve the readers' understanding of the molecular and cellular processes involving periodontal regeneration essential for designing comprehensive therapeutic approaches.

## 1 INTRODUCTION TO REGENERATIVE MEDICINE IN PERIODONTOLOGY

The periodontal complex involves the hard and soft tissues which support dentition. Given its significant roles in force transduction, providing sensory information about tooth position, facilitating jaw reflexes during tooth movement, and resistance to mechanical forces, and the prevalence of periodontal disease, regeneration of the periodontium is a significant and complex clinical challenge (Zhao, Volponi, Caetano, & Sharpe, 2020). The goal of tissue engineering and regenerative medicine is to repair and replace damaged tissues in the course of the disease, to restore physiologic function (Hoffman, Khademhosseini, & Langer, 2019; Langer & Vacanti, 1993, 2016). In the case of periodontal tissues, the field of periodontology has made significant progress in translating periodontal regeneration techniques to the patient care (Galli, Yao, Giannobile, & Wang, 2021; Giannobile & McClain, 2015; Menicanin, Hynes, Han, Gronthos, & Bartold, 2015). While considerable progress has been made in areas of alveolar bone and gingival soft tissue regeneration, these treatments have had limited success in recapitulating a regenerated periodontal ligament's (PDL) structural and functional aspects. This review does not cover perspectives on the potential regeneration of teeth, but we encourage interested readers to reference El Gezawi, Wölfle, Haridy, Fliefel, and Kaisarly (2019) and W. Zhang and Yelick, (2021). This review aims to highlight advances in periodontal regeneration, emphasizing the need for future development to increase the predictability and outcomes of PDL regeneration informed by recent advances in understanding its developmental origins.

**FIGURE 1:**
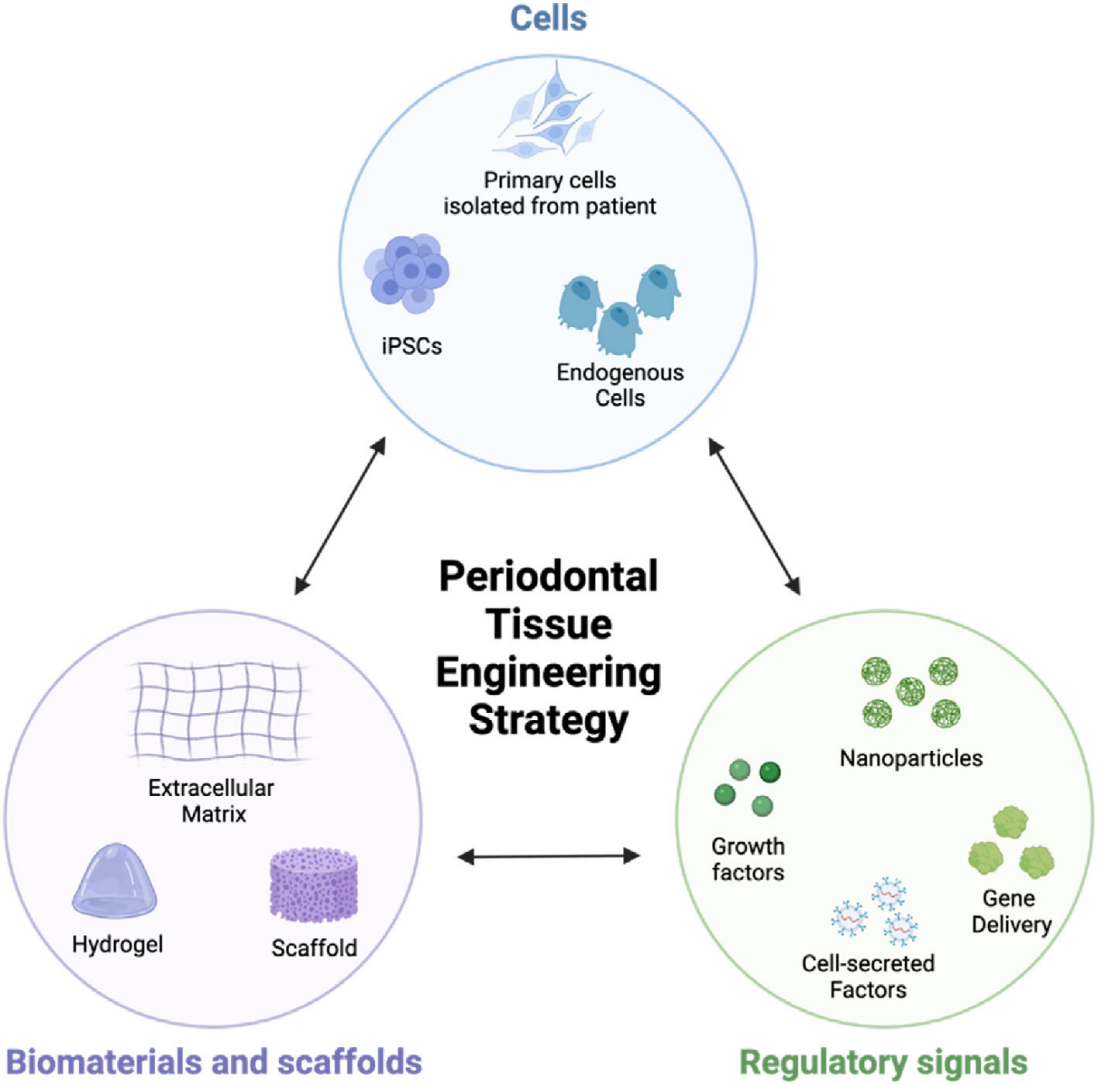
The tissue engineering triad illustrates the three critical design criteria for tissue engineering strategies. Figure prepared with Biorender

Tissue engineering aims to recapitulate three‐dimensional (3D) tissue structure by bringing together cells capable of regeneration with an environment adequate for tissue neogenesis, including both physical and chemical inductive signals (Chan & Leong, 2008). These crucial components are referred to as the tissue engineering triad and are essential in regenerative strategies' design and clinical implementation (Figure 1).

- Biomaterials Scaffold: Biomaterial scaffolds provide a 3D environment that organizes cells in three dimensions (Swanson & Ma, 2020); recent advances in biomaterials engineering and mechanobiology point to the role of biomaterials' physical morphology and mechanical properties as crucial design motifs in determining the trajectory of cells in engineered microenvironments (Gupte et al., 2018; Naqvi & McNamara, 2020; Swanson et al., 2021; Y. Zhang et al., 2017). Biomaterials must be biocompatible to prevent host rejection and biodegradable at a rate that facilitates replacement of the degrading scaffold with new tissue without loss of engineered tissue integrity (Langer & Vacanti, 1993, 2016). Biomaterial scaffolds may be combined with drug delivery systems for sustained and controlled delivery of inductive signals to the tissue defect compartment.
- Regenerative cell population: Cells capable of regeneration may be endogenous cells that migrate to a defect site or may be transplanted (Langer & Vacanti, 1993). Several kinds of stem cells have been explored as candidates for their usefulness in regenerating dental tissue, including dental pulp stem cells (Rubins, Tolmie, Corsig, Kerr, & Kim, 2014), stem cells from the apical papilla (Kang, Fan, Deng, He, & Huang, 2019), dental follicle precursor cells (Guo et al., 2012), PDL stem cells (Queiroz et al., 2021), alveolar bone stem cells (Tan et al., 2009), and stem cells from human exfoliated deciduous teeth (SHED) (Gao et al., 2018).
- Inductive signals: Inductive signals increase the predictability and specificity of regenerative trajectories (Ren, Zhao, Lash, Martino, & Julier, 2020). Most often, inductive signals are growth factors and morphogens but may also be small molecule and peptide therapeutics or genes and other bioactive molecules. Similarly, inductive signals may come from cells surrounding a defect or secreted by transplanted or newly migrated cells (Kim, Hong, & Son, 2016). Biomaterial matrices may provide tissue inductive signals by promoting specific differentiation fates of cells through engineered physical, chemical, and mechanical stimuli (Rambhia & Ma, 2015).

This highly interdisciplinary approach to treating disease involves recapitulating tissue and organ formation and relies significantly on recent findings from developmental biology, stem cell biology, advanced tissue culture techniques, and biomaterials engineering.

## 2 CLINICAL SIGNIFICANCE OF THE PDL IN HEALTH AND DISEASE

**FIGURE 2:**
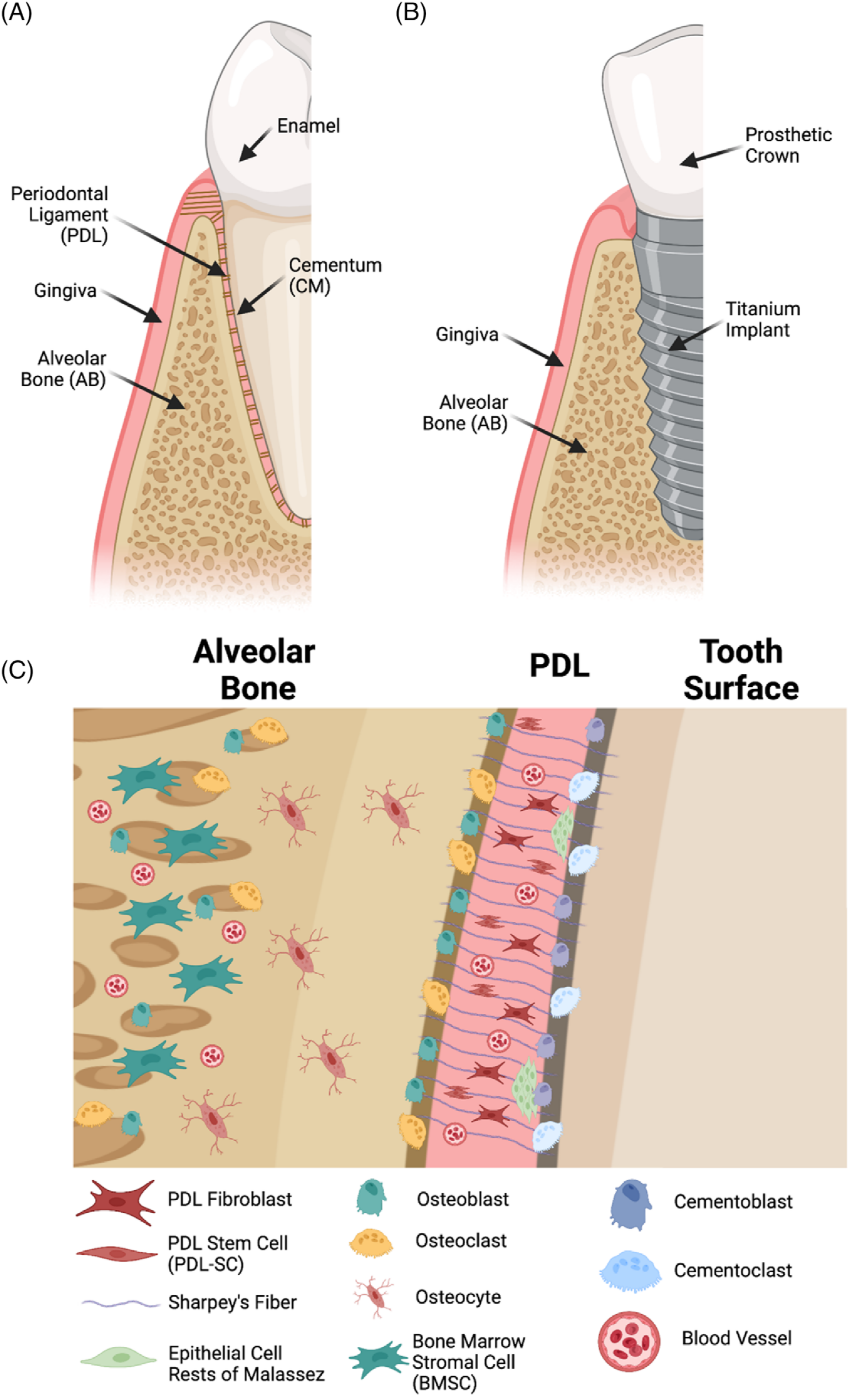
Schematic overview of periodontal tissues in the case of native dentition (a) and dental implants (b). The physiologic periodontium involves a complex milieu of cell types between alveolar bone, the periodontal ligament, and cementum (c). Figure prepared with Biorender

The PDL is a dynamic and biomechanically active craniofacial fibrous joint that provides a direct interface between teeth and the supporting alveolar bone (Figure 2a) (Chiego, 2018; Ten Cate, 1998). The PDL serves to attach teeth to the alveolar bone. In doing so, it facilitates tooth displacement within the bony socket where teeth reside and acts to dampen and distribute cyclic masticatory forces. It sustains tissue remodeling in response to mechanical forces in the cementum and bone (McCulloch, Lekic, & McKee, 2000).

The PDL comprises bundles of parallel collagen fibers and elastin, which are highly ordered to withstand, respond to, and transmit masticatory loads (S. H. Liu, Yang, Al‐Shaikh, & Lane, 1995). The PDL comprises collagen Type I but also contains Types V and VI, chondroitin sulfate, proteoglycans, fibronectin, tenascin, and undulin. These fibers insert into the alveolar bone and cementum as Sharpey's fibers, which serve as anchoring points. Interestingly, McKee, Zalzal, and Nanci (1996) demonstrated that Sharpey's fibers accumulate high levels of osteopontin at their insertion site in alveolar bone, but not in cementum, among other non‐collagenous proteins with distinct spatial organization within the PDL. The PDL is richly vascularized with blood vessels that communicate with those from the gingiva, alveolar bone, and apical region of teeth, with a standard pattern of occlusal‐apical orientation (Masset, Staszyk, & Gasse, 2006). The PDL also contains dense innervations by myelinated nerve fibers near collagen fibers and the alveolar bone (Y. Huang, Corpas, Martens, Jacobs, & Lambrichts, 2011).

Based on the observation that the PDL is maintained throughout the highly variable applied loads experienced in normal masticatory function, the PDL requires a highly effective system for sensing, maintaining, and adapting its role. Periodontal disease involves the loss of tooth‐supporting structures; its hallmark feature is the loss of alveolar bone (Tonetti, Greenwell, & Kornman, 2018). Along with alveolar bone loss is the loss of the bone‐integrated support structure—the PDL. Periodontitis is the 11th most common disease worldwide and is identified by the US Surgeon General's Report on Oral Health as a critical challenge to human health and the disease (Kassebaum et al., 2017). Therefore, periodontal disease represents a significant healthcare burden (Botelho et al., 2022), and developing regenerative treatments is a meaningful and vital opportunity. Loss of the PDL may also result in a manifestation of systemic disease, as reviewed by Albandar, Susin, and Hughes (2018). These systemic diseases largely influence periodontal inflammation, notably obesity, osteoporosis, diabetes mellitus, and HIV infection. Independent of periodontitis, odontogenic tumors and neoplasms of periodontal tissue, scleroderma, Langerhans cell histiocytosis, and giant cell granulomas can result in loss of periodontal tissue.

Dental implants replace dentition lost to trauma or disease as an implantable prosthetic. In contrast to native dentition, titanium dental implants directly integrate with the alveolar bone (Figure 2b) (Buser, Sennerby, & De Bruyn, 2000). Osseointegration has been demonstrated to be critical for implant stability and long‐term clinical success (Rutkowski, 2018). Collagen fibers in the surrounding alveolar bone are oriented parallel to the implant surface. They do not attach to the implant, unlike PDL fibers oriented perpendicular to Sharpey's fibers attachments into the cementum and bone (Tete, Mastrangelo, Bianchi, Zizzari, & Scarano, 2009; Traini, Degidi, Strocchi, Caputi, & Piattelli, 2005). We believe this represents an exciting opportunity for innovation, which will be discussed in further sections.

The PDL contains and interfaces with several distinct cell populations (Figure 2c). The predominant cell type is the fibroblasts (McCulloch & Bordin, 1991). Fibroblasts are principally responsible for collagen turnover and maintenance within the PDL (Everts, van der Zee, Creemers, & Beertsen, 1996). PDL fibroblasts activated by mechanical forces secrete plasminogen activator and matrix metalloproteases, as well as their inhibitors, cytokines (i.e., PGE‐2), and interleukin‐6 (Lekic, Rajshankar, Chen, Tenenbaum, & McCulloch, 2001). There is spatial, developmental, and functional heterogeneity among PDL fibroblasts (Freeman & ten Cate, 1971; Groeneveld, Everts, & Beertsen, 1993; McCulloch & Bordin, 1991).

Alveolar bone and cementum interface with the PDL. Osteoblasts and osteoclasts on the surface of alveolar bone have anabolic and resorptive functions, respectively, are responsible for bone turnover and adaptation, and are also involved in orthodontic tooth movement (N. Jiang et al., 2016). Similarly, cementoblasts and cementoblasts in the cementum are responsible for anabolic and resorptive functions, respectively. Epithelial cell rests of Malassez are also found in the PDL and are quiescent epithelial remnants from Hertwig's epithelial root sheath, involved in the formation of tooth roots (Shinmura, Tsuchiya, Hata, & Honda, 2008). They play a role in the cementum and enamel repair (Tsunematsu et al., 2016). Endothelial cells form the lining of blood vessels.

## 3 PERSPECTIVE ON PDL REGENERATION

Following insult by trauma or disease, the ideal clinical outcome would be the regeneration of the periodontal support apparatus, including alveolar bone, cementum, and PDL. New attachment, therefore, involves the regeneration of the principle fibers which compose the PDL *and* their reattachment into newly formed cementum on the root surface. This process, performed partly by PDL cells, competes with apical migration of the pocket‐lining junctional epithelium (Wikesjo, Sigurdsson, Lee, Tatakis, & Selvig, 1995). Nyman et al. were among the first to demonstrate that a new PDL can be established on a previously diseased root surface (Nyman, Lindhe, Karring, & Rylander, 1982). Their seminal study in a human patient showed that cells originating from the PDL might migrate coronally to a root surface previously exposed to a periodontal pocket to deposit new cementum and attachment fibers when adequately isolated from the sulcular epithelium with a cellulose acetate membrane. Furthermore, they demonstrated that the formation of new connective tissue attachment is not necessarily accompanied by coronal regeneration of alveolar bone.

The PDL contains a self‐renewing progenitor cell population, identified as the PDL stem cell (PDL‐SC), found in perivascular spaces of the periodontium (McCulloch, Barghava, & Melcher, 1989; McCulloch & Melcher, 1983; Seo et al., 2004). This stem cell population is critical to the PDL's ability to adapt and self‐renew throughout life. PDL stem cells obtained from mature PDL of erupted teeth possess similar stem cell properties to mesenchymal stem cells (MSCs) (Kaku et al., 2012). Their molecular characteristics are summarized by Zhu and Liang (2015). These cells are characterized by a rapid turnover rate and distinct localization near blood vessels in the PDL and are capable of osteogenic, cementogenic, and fibroblastic differentiation fates (Palmer & Lumsden, 1987). Compared to bone marrow mesenchymal stem cells (BMSCs) often used in preclinical models of craniofacial regeneration, both BMSC and PDL‐SCs are capable of robust bone regeneration, but only PDL‐SCs show a favorable effect on PDL formation (Yan, Yang, Jansen, de Vries, & van den Beucken, 2015). Their study is significantly important to PDL regeneration and is an area of active investigation.

PDL‐SCs are a key cell source to harness in PDL regeneration. Importantly, they are present in human adults throughout life. However, their number decreases with patient age (Zheng et al., 2009) and can be expanded ex vivo (Seo et al., 2004) as well as recovered from cryopreservation (Seo et al., 2005). In the context of autologous use, PDL‐SCs from adults older than 41 years demonstrated decreased regenerative capacity. Growth factor treatment, optimized growth factor administration, or biomaterials capable of stem cell expansion are exciting. Allogenic use, rather than autologous, may also be possible. MSCs from the gingiva may be more easily accessible for cell isolation when tooth extraction is not indicated. They have been demonstrated to regenerate cementum, alveolar bone, and PDL in a canine model (Seo et al., 2005) and may be more resistant to the effects of inflammation than PDL‐SCs (Yang et al., 2013). In vitro models of both PDL‐SCs and GMSCs will allow for a high throughput screening platform of nanoparticle, small molecule, and protein therapeutics and provide important insight into their molecular behavior.

Yan, Yang, et al. (2015) summarized the state of periodontal tissue regeneration in preclinical animal models in a 2015 meta‐analysis, which provided significant insight into regenerative outcomes and cell sources. They included 39 studies fitting their meta‐analysis criteria. PDL‐derived cells and BMSCs are equally efficacious in inducing new bone formation; differences in cementum formation capacity were not statistically significant. Interestingly, only PDL‐SCs show a favorable effect on PDL formation. Like BMSCs, PDL‐SCs have been demonstrated to possess unique immunomodulatory properties, which may be advantageous for cell transplantation therapies (Wada, Menicanin, Shi, Bartold, & Gronthos, 2009), summarized by Wada et al.

Feng et al. (2010) provided an early report in which periodontal defects were treated with autologous PDL progenitor population. PDL‐SCs were isolated from extracted third molars; the PDL tissue was separated from the tooth, and cells were cultured on a calcite surface resulting in a cell sheet. The calcite/PDL‐SC sheet was inserted into a mucoperiosteal flap following debridement. While limited by patient number (n = 3), the authors described significant decreases in probing depth and mobility and increased clinical attachment and radiographic bone fill. F.‐M. Chen et al. (2016) report an early small‐scale randomized clinical trial demonstrating the utility of autologous PDL‐SCs delivered in combination with a bone xenograft material (Bio‐oss) to regenerate alveolar bone height and clinical attachment with no adverse events. These trials serve as preliminary evidence for the potential of PDL‐SCs as valuable agents of PDL regeneration in the broader context of periodontal regeneration. We will summarize the current state of the art in periodontal regeneration and revisit this evidence to date, considering its future development.

Significant excitement in periodontal regeneration has been generated in both preclinical and clinical literature. Multiple stem cell technologies have been successfully validated in human clinical trials to regenerate dental/oral tissues. Furthermore, in various small sample studies, clinicians' attitudes (Aiyegbusi et al., 2020; y Baena, Casasco, & Monti, 2022) and medical and dental students (Burdick, Mauck, Gorman, & Gorman, 2013) are positive toward cell‐based regenerative technologies. These surveys also demonstrate poor knowledge of the use of stem cells and a need for significant clinician education to enable their efficient clinical adoption (y Baena et al., 2022). Interestingly, the opinions of dental hygienists and assistants, among other clinical auxiliary staff, have not been well reported. These individuals are key players in the patient care team and will likely play a significant role in the clinical workflow related to stem cell‐based technology. Furthermore, patient education and public perception will require appropriate education on various aspects of the new medical technology (Aiyegbusi et al., 2020). Biomaterials and growth factors that take advantage of endogenous cell sources, rather than cell transplantation, may represent a more near‐term technology achievement in the periodontal regeneration (Burdick et al., 2013); mechanical debridement procedures highlight the endogenous regenerative capacities of the periodontium, which exogenous factors can further augment.

## 4 CURRENT PROGRESS IN PERIODONTAL REGENERATION

Scaling and root planing (SRP) remains a gold standard non‐surgical treatment for periodontal defects by the mechanical removal of disease‐causing agents (Herrera, 2016). The primary goal in removing subgingival calculus, biofilm deposits, and diseased cementum is to create a root surface capable of reattachment with periodontal tissues. SRP, along with oral hygiene instruction for home care and patient compliance, results in favorable clinical outcomes in a non‐surgical approach to the treatment (Brayer, Mellonig, Dunlap, Marinak, & Carson, 1989; Herrera, 2016). The effectiveness of SRP becomes limited by instrument limitations (Stambaugh, Dragoo, Smith, & Carasali, 1981), where curet efficiency decreases below 3.73‐mm pocket depth and reaches a limit of ~6‐mm depth. Beyond a critical probing depth of 5.4 mm, open flap debridement, a form of surgical periodontal therapy, may be preferred (Heitz‐Mayfield & Lang, 2013; Lindhe, Socransky, Nyman, Haffajee, & Westfelt, 1982).

**FIGURE 3:**
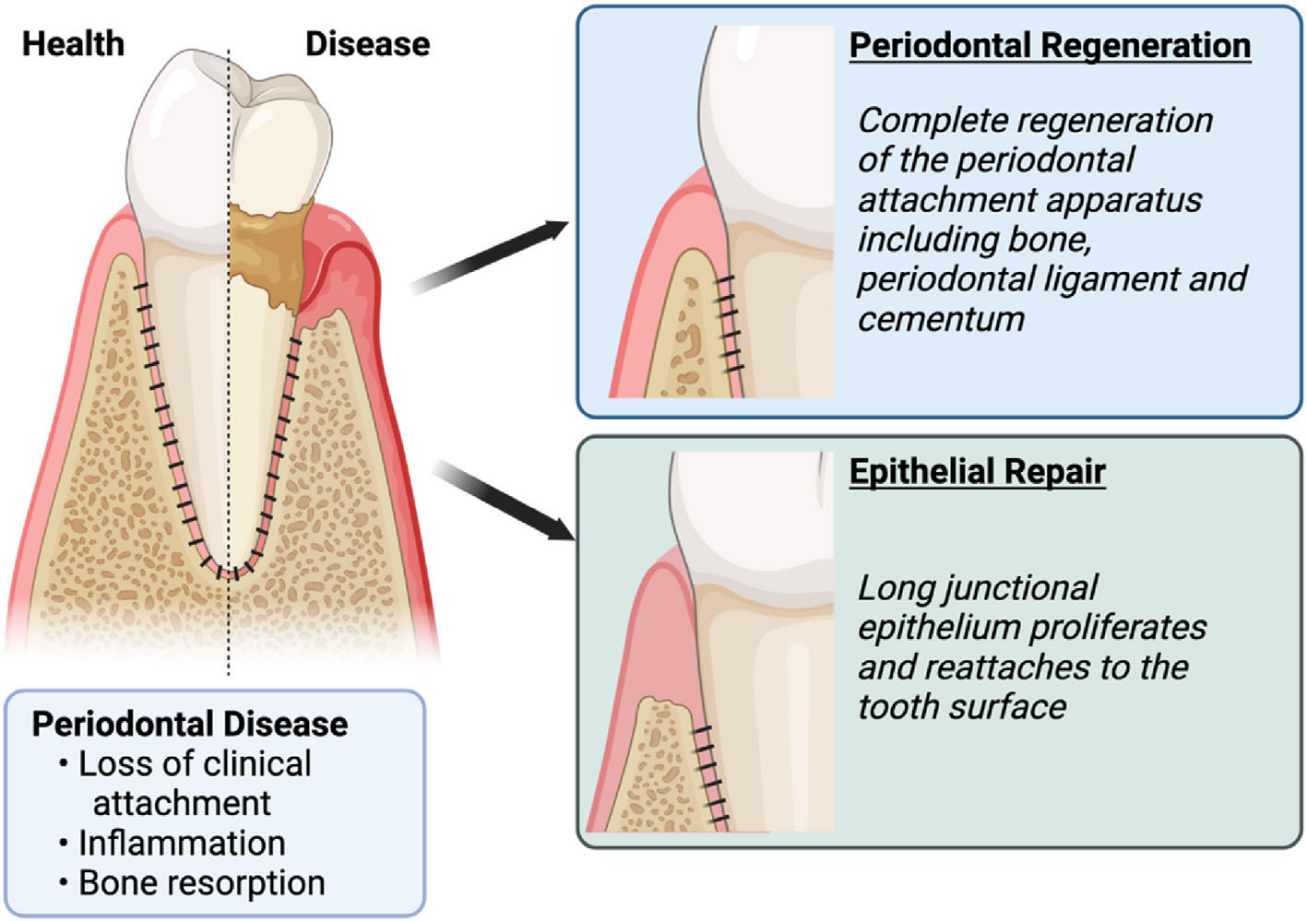
Therapeutic outcomes in the treatment of periodontal disease include periodontal regeneration and epithelial repair. Figure prepared with Biorender

The goals of periodontal surgical therapy are to treat periodontal disease or modify the morphological status of the periodontium, enabled by surgical access to deeper defects (Comprehensive Periodontal Therapy, 2011). Periodontal regeneration aims to facilitate the formation of new bone, cementum, and a functionally oriented PDL at a site deprived of its initial attachment apparatus (Giannobile & McClain, 2015). (Figure 3). Systematic reviews and consensus reports from the American Academy of Periodontology (AAP) are published in the *Journal of Periodontology*, including practical application reports. This review is not intended as a comprehensive clinical review. Still, it serves to highlight advances in tissue engineering specifically to the intersection of developmental biology and regeneration, and we encourage interested readers to consult the AAP.

Guided bone regeneration seeks to fulfill the specific goal of bone regeneration in preparation for dental implant placement without implicit concern for the PDL. These procedures may include alveolar ridge reconstruction, sinus floor augmentation, and resurrection of preimplant osseous defects. Guided tissue regeneration (GTR) seeks to reconstitute lost periodontal structures through different tissue responses, allowing for a renewal of the periodontal attachment apparatus, including bone, PDL, and cementum (Melcher, 1976; Wolff, 2000). Clinical indications of GTR are summarized by Wolff (Wolff, 2000). In addition to barrier membranes described above which allow for selective repopulation of the root surface, growth factors play a critical role in development and regeneration. They are a vital component of tissue engineering therapies.

In vitro studies of PDL‐SCs demonstrate their responsiveness to exogenous growth factor administration and secreted factors from neighboring cells found in the periodontium. Bone morphogenic proteins BMP‐2 and BMP‐7 and vascular endothelial growth factor increase the osteogenic capacity of PDL‐SCs (Hakki et al., 2014; Maegawa et al., 2007). Transforming growth factor beta (TGF‐β1) and connective tissue growth factor, and fibroblast growth factor (FGF) improve the fibroblastic differentiation (Fujii et al., 2010; Yuda et al., 2015). FGF‐2 promotes PDL‐SC proliferation but reverses the effects of BMP‐2 on osteogenic differentiation when co‐administered with J. H. Lee, Um, Jang, and Seo (2012). Their sequential administration may be optimized to encourage both PDL‐SC expansion then osteogenic differentiation. FGF‐2 administration to PDL‐SCs and MSCs upregulates the expression of BMP receptor‐1B (BMPR‐1B), enhancing the osteogenic effects of BMP‐2 (Nakamura et al., 2005). Growth factors may also play an essential role in maintaining the stemness of PDL‐SCs in treatment and long‐term in vitro culture.

Several growth factor therapies have received FDA approval in various indications of periodontal regeneration, and their clinical use has been validated in on‐label and off‐label uses. A systematic review from the 2015 AAP Regeneration Workshop highlights that biologics are generally comparable and compatible with allograft and GTR and superior to open flap debridement procedures for treating infrabony defects. These positive outcomes can be maintained over long periods, greater than 10 years (Kao, Nares, & Reynolds, 2015; Reynolds et al., 2015). Table 1 provides an overview of biologics currently used in clinical periodontal regeneration.

**TABLE 1:** Growth factors used in clinical periodontal regeneration

Biologic	Trade name	Source	FDA‐approved indications	Biologic significance	Mechanism	Clinical trials
Enamel matrix derivative	Emdogain (Straumann)	Swine tooth germ (dispersed in propylene glycol alginate matrix) containing enamel matrix proteins, largely amelogenins	Intrabony defects due to moderate or severe periodontitis Mandibular class II furcation defects Gingival recession defects in conjunction with surgical coverage procedures Minimally invasive surgical technique in esthetic zones to optimize tissue heigh for infrabony defects	Enamel matrix proteins are secreted by Hertwig's epithelial root sheath during development of the enamel organ (Kornman, Giannobile, & Duff, 2017)	Recruitment of cementoblasts to form neocementum [154], fibroblasts [155], osteoblasts [156], and PDLSCs [157, 158] leading to mineralization. EMD remains on the root surface for up to 4 weeks [159]	Infrabony defect [Miron et al., 2013, 160–162] Furcation defect [163, 164] Gingival recession [165–167]
Platelet derived growth factor	GEM‐21S (Lynch Biologics)	rh‐PDGF‐ββ	Intrabony periodontal defects Furcation periodontal defects Gingival recession associated with periodontal defects Note: Approved for use in combination with beta‐tricalcium phosphate biomaterial	PDGF is secreted by platelets upon activation and triggers fibroblast proliferation, migration, and differentiation [168, 169]	Migration and proliferation of osteoblasts, PDL cells and cementoblasts. Stimulation of angiogenesis (Wikesjo, Sorensen, Kinoshita, Jian Li, & Wozney, 2004)	Intrabony periodontal defect [94, 170, 171] Furcation defects [170, 172, 173] Soft tissue augmentation [174, 175]
Bone Morphogenic protein	Infuse (Medtronic)	rh‐BMP2	Sinus augmentation Localized alveolar ridge augmentation for defects associated with extraction sockets (socket preservation) Note: Approved for use in combination with carrier/scaffold such as adsorbable collagen sponge	BMPs play crucial roles in all organ systems. Endogenous BMP signaling is tightly regulated and involved in the skeletal development [176, 177]	BMP‐2 stimulates osteoblast chemotaxis, differentiation, alkaline phosphatase activity, and osteocalcin synthesis/mineralization [178, 179]	Sinus augmentation: [180–182] Alveolar ridge + socket preservation: [183, 184]
Platelet rich plasma (PRP)	N/A	Autologous blood with concentrated platelets >250,000 platelets/μl	The FDA does not regulate or approve of PRP treatments; the kits and devices used to prepare PRP require clearance [185]	Circulating growth factors and other inductive molecules to promote healing are concentrated and used autogenously	The therapeutic effects of PRP are attributed to “bioactive factors” [186, 187]. A discrete mechanism of action is not defined	A comprehensive review of PRP in periodontal regeneration of intrabony defects is summarized by Rosello‐Camps et al. [188]

The summary in Table 1 demonstrates significant interest and development in growth factor therapeutics used in periodontal regeneration. These developments are informed by decades of investigational research into inductive factors which play pivotal roles in periodontal and dentoalveolar development and maintenance. Significant progress has been made in their routine clinical implementation, but not without limitations:

- Enamel matrix derivative (EMD): Initial studies suggested up to three times greater defect fill when compared to open flap debridement alone (Heijl, Heden, Svardstrom, & Ostgren, 1997) and significant clinical attachment gain (Miron et al., 2016). Other trials have failed to demonstrate heterogeneity in its clinical effect (Esposito, Grusovin, Papanikolaou, Coulthard, & Worthington, 2009; Miron et al., 2013) or lack of effect compared to non‐biologic GTR protocols in single intrabony defects (Gutierrez, Mellonig, & Cochran, 2003; Mombelli, Brochut, Plagnat, Casagni, & Giannopoulou, 2005). Various studies have demonstrated heterogeneity in regenerative outcomes due to adsorption and efficient delivery of EMD. A new commercial product, Osteogain (Straumann), is in development which aims to improve the combination of EMD and bone grafting materials (J. Chen et al., 2015; Miron et al., 2012).
- Platelet‐derived growth factor (PDGF): rhPDGF‐ββ combined with a bioresorbable material, beta‐tricalcium phosphate, enabled its delivery and was the first entirely synthetic product approved by the FDA to treat periodontal‐related defects (Nevins et al., 2005). Tavelli et al. (Wikesjo et al., 2004) provides an in‐depth review of its clinical potential, which suggests strong evidence for its efficacy in regenerating infrabony defects when used in conjunction with a bone matrix. Various clinical and human histologic studies have demonstrated evidence of new bone, PDL, and cementum formation following rhPDGF‐ββ administration.

In addition to growth factors, significant progress in biomaterials engineering for periodontal regeneration has been made and adopted into widespread clinical practice. Given that patient behaviors appreciably influence clinical outcomes and surgical approach rather than tooth and defect characteristics taking advantage of the inductive features of biomaterials may significantly enhance regenerative effects (Cochran et al., 2015; Kao et al., 2015; Reynolds et al., 2015). The surgical technique also undoubtedly plays a role in their clinical success.

A significant reason for using biomaterials is to facilitate the delivery of biologic agents to the defect site. These materials simultaneously provide an artificial matrix to accelerate wound healing, repair, and regeneration (Swanson & Ma, 2020). Biomaterials can be further engineered to enable delivery with sensitivity to spatial and temporal requirements of biologic agents and healing processes. Somerman (2010) describes that while agents on the market (Gem21S, Emdogain, FGF‐2) have demonstrated promise in various aspects of periodontal regeneration, there is room for improvement in the predictability of their ability to achieve sufficient regeneration, which may require combinations of growth factors with controlled deliveries. Herein we will discuss recent advances in growth factors, biomaterial delivery mechanisms, and areas for continued investigation.

## 5 DEVELOPMENT OF NEXT‐GENERATION REGENERATIVE THERAPEUTICS

A literature review illustrates significant progress in periodontal regeneration in the last 30 years, and gaps for improvement have been identified. Regeneration of the PDL requires the concerted, synchronized activity of multiple cell types and molecular processes. We believe that an intimate understanding of the development and physiologic maintenance of the PDL and surrounding structures is a prerequisite to developing next‐generation regenerative protocols and technologies. The ideal regenerative therapeutic will satisfy the following regenerative criteria:

- cementoblastogenesis on the tooth root surface;
- oblique insertion of PDL fibers into cementum and alveolar bone;
- vital supporting bone.

We believe the key challenges in achieving these regenerative goals are the management of vascularization in periodontal tissues, the potential for microbial contamination into the defect, and masticatory forces present during healing.

### 5.1 Next‐generation growth factors for periodontal regeneration

Next‐generation growth factor therapeutics must consider this complexity of the periodontium and will draw inspiration from an improved understanding of its physiologic development. We will highlight advances in growth factor therapeutics that have demonstrated promising in vitro and preclinical in vivo results, warranting further development.

BMP‐6: BMP‐6 is similar in structure to BMP 5 and 7. It is responsible for osteoblast differentiation. In a study of BMP‐6 and collagen sponge carriers applied to periodontal fenestration defects in rats, complete osseous healing occurred in BMP‐6‐treated animals after 4‐weeks (K. K. Huang, Shen, Chiang, Hsieh, & Fu, 2005).

GDF‐7: In a pilot study, the potential of growth and differentiation factor‐7 (GDF‐7)/BMP‐12 to stimulate PDL formation was evaluated in a supra‐alveolar periodontal defect model. This study suggested that GDF‐7 has a significant potential to support the regeneration of the PDL (Wikesjo et al., 2004).

GDF‐5: GDF‐5 (BMP‐14) is another member of the TGF‐β superfamily of interest for the periodontal regeneration (Moore, Dickinson, & Wikesjö, 2010). BMP‐14 is expressed in developing periodontal tissues (Morotome, Goseki‐Sone, Ishikawa, & Oida, 1998) and primordial cartilage in early limb development (Francis‐West et al., 1999; Storm & Kingsley, 1999). GDF‐5 has a high binding affinity to BMPR1B, BMPR2 (Nishitoh et al., 1996), and Activin Type II receptors (Klammert et al., 2015). Human clinical trials have demonstrated its efficacy in periodontal regeneration: intrabony defect (Stavropoulos, Windisch, et al., 2011) and sinus augmentation (Koch, Becker, Terheyden, Capsius, & Wagner, 2010; Stavropoulos, Becker, et al., 2011). GDF‐5 was under development by Scil Technology, GmbH, as MD05. Scil Technology, GmbH is no longer pursuing clinical development, and the asset was licensed to Medtronic for development (Emerton et al., 2011). Recent evidence in protein engineering suggests the GDF‐5 mutant BB‐1 may have enhanced osteoinductive capacity in a large animal model (Gunnella et al., 2018).

Teriparatide (Forteo, Eli Lilly, Inc.): Teriparatide is a synthetic form of human parathyroid hormone (PTH), which stimulates new bone formation. In the first human clinical trial with teriparatide in periodontal healing, 40 patients with periodontitis were treated with teriparatide or placebo once daily for 6 weeks following open flap debridement. Treatment with teriparatide led to a greater resolution in osseous defects and improved clinical attachment, where even short dosing of the drug had significant long‐term effects (Bashutski et al., 2010; Grover, Luthra, & Maroo, 2013).

FGF‐2: FGF‐2 promotes the proliferation of osteoblasts and fibroblasts (Murakami, 2011). FGF‐2 also possesses angiogenic and mitogenic activity on mesenchymal cells within the PDL (Suarez‐Lopez Del Amo, Monje, Padial‐Molina, Tang, & Wang, 2015). While not FDA‐approved for its use in the US, FGF‐2 is approved for human clinical use in Japan in a topical formulation for regenerating periodontal tissues destroyed by periodontitis Kitamura et al. (2011) (Kaken Pharmaceuticals). It demonstrates significant regenerative potential compared to vehicle treatment but has not been well‐studied in head‐to‐head comparisons with other growth factors (Cochran et al., 2016).

Exosome therapy: Exosomes are a subset of extracellular vesicles, lipid‐bound nanoparticles secreted by cells and containing various signaling molecules, including DNAs, RNAs, and proteins (Narayanan, Huang, & Ravindran, 2016; Swanson & Mishina, 2022). Exosomes have been thought to be nature's endogenous nanoparticle delivery platform, and in particular, their miRNA cargo and growth factor are of significant interest (Swanson, Gong, et al., 2020; Swanson & Mishina, 2022; Swanson, Zhang, et al., 2020). Recently, human bone marrow stromal cell‐derived exosomes were demonstrated as a promising therapeutic in a preclinical rat periodontitis model to reduce tissue destruction and immune cell infiltration (Yue et al., 2022). The authors identified enrichment miRNAs associated with negative regulation of inflammatory response and increased protein abundance of factors including FGF‐6, insulin growth factor (IGF‐1), and interleukins: IL‐1ra, IL‐16, and IL‐3 concentrated in exosomes.

### 5.2 Next‐generation signal delivery for periodontal regeneration

Small molecule drugs and protein cargo can be encapsulated by biodegradable materials through double emulsion technologies yielding bioactive molecules encapsulated within a biodegradable polymeric shell. Biodegradable biomaterials, which degrade over time in the body, are adequate to allow for the sustained release of drugs and inductive factors. As the material degrades due to hydrolysis in the physiologic environment, factors escape entrapment and are exposed within the defect site (Langer & Vacanti, 2016; Ma, 2008). PDGF‐ββ (Wei, Jin, Giannobile, & Ma, 2006) has been demonstrated to tolerate encapsulation in a poly(glycolic‐co‐lactic acid) PLGA polymeric vector, maintaining their biologic efficacy following encapsulation and release. PDGF‐ββ encapsulated in microspheres is easily attached to nanofibrous tissue engineering scaffolds (Wei et al., 2006). Parathyroid hormone and exosomes each represent examples of biologics requiring suitable delivery platforms to enable their delivery. Parathyroid hormone (Teriparatide) requires a pulsatile administration to induce bone formation, given the observation that continuous PTH exposure results in bone resorption. Dang and others developed an innovative mechanism for the preprogrammed long‐term pulsatile delivery of PTH, allowing for anabolic bone regeneration (Dang, Koh, Danciu, McCauley, & Ma, 2017; Dang, Koh, Jin, McCauley, & Ma, 2017; X. Liu, Pettway, McCauley, & Ma, 2007). Lipid nanovesicle exosomes are much larger than protein biologics and require stabilization of their lipid membrane, internal protein, and nucleic acid cargo. Swanson and colleagues demonstrated the first controlled release system for the efficient encapsulation and controlled release of exosomes and extracellular vesicles in a nanoparticle system, allowing for their sustained release (Swanson, Gong, et al., 2020; Swanson, Zhang, et al., 2020). The release kinetics of encapsulated exosomes is tunable based on the biomaterial composition.

**FIGURE 4:**
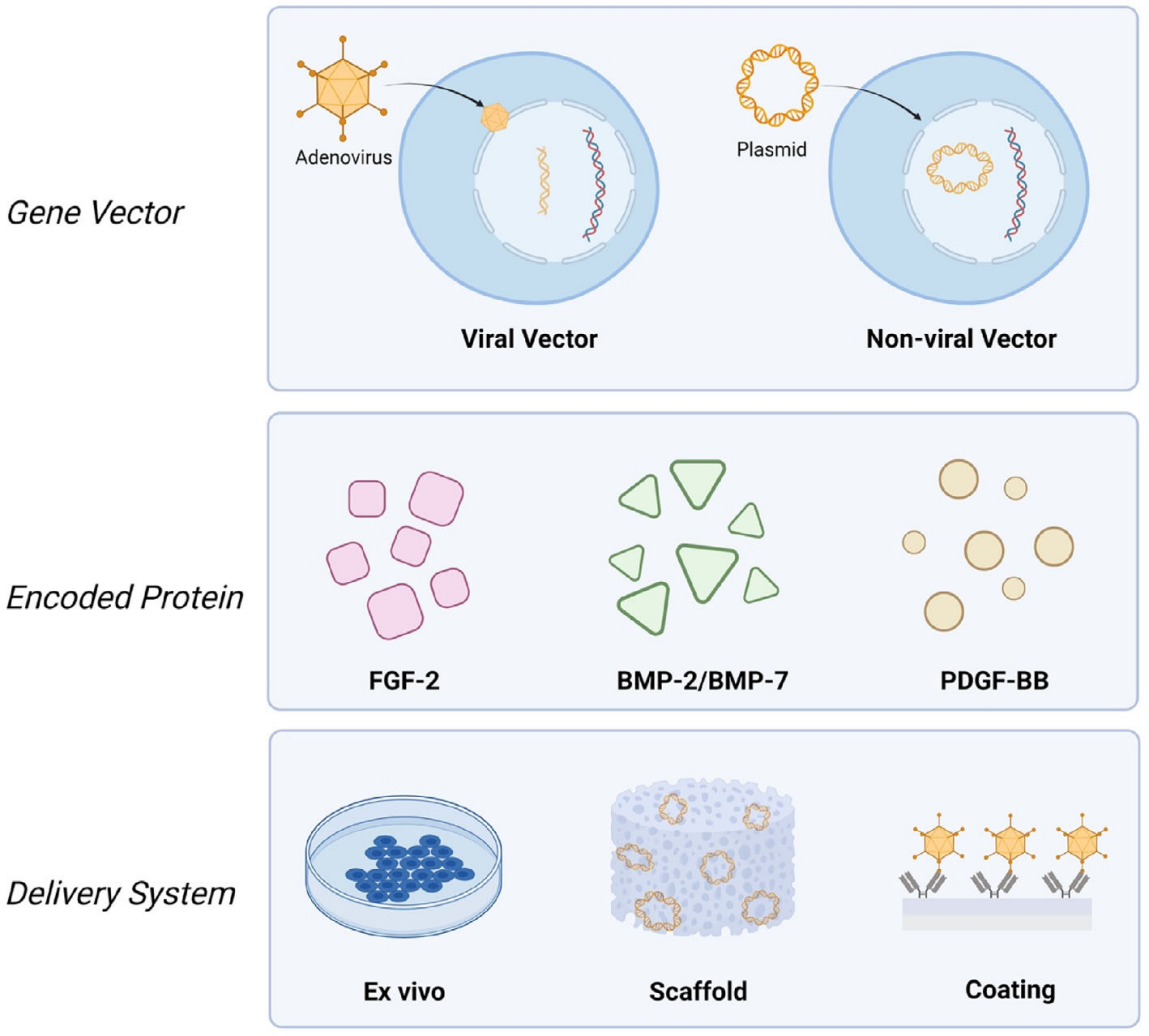
Schematic representation of gene delivery technologies and their various embodiments which may be used in periodontal regeneration. Figure made with Biorender

To regenerate hierarchically structured tissues, such as periodontium, it is essential to trigger appropriate and precise signals to direct cell populations to form appropriate tissue types in the correct anatomical locations (Gonzalez‐Fernandez et al., 2019). The specific spatial or temporal delivery of signaling molecules, such as growth factors, can be challenging. To overcome those limitations, various gene therapies have been developed specifically for periodontal diseases, allowing for sustained synthesis and secretion of one or multiple growth factors by genetically modifying cells (Figure 4) (Galli et al., 2021).

Viral vectors used for gene delivery include adenovirus (Ad), adeno‐associated virus, lentivirus, retrovirus, and baculovirus, each of which has its advantages and disadvantages (Galli et al., 2021). Ad has been frequently used in periodontal regeneration because of several unique features compared with other viral vectors: (1) high transfection efficiency in both dividing and non‐dividing cells; (2) does not induce apparent phenotypic changes; and (3) does not integrate into host genome (Gu et al., 2004). Dunn et al. delivered Ad‐BMP‐7 using a collagen matrix around titanium dental implants to treat peri‐implant osseous defects and revealed the sustained transgene expression for up to 10 days at the osteotomy sites (Dunn et al., 2005). In addition to Ad‐BMP‐7, Ad‐PDGF‐B used in large periodontal osseous defects has been demonstrated safe with improved alveolar bone and cementum regeneration for possible use in human clinical studies (Chang et al., 2009; Jin, Anusaksathien, Webb, Printz, & Giannobile, 2004).

Based on the results from previous studies and the architecture of periodontium consisting of PDL fibroblasts and osteoblasts, various groups have developed localized and sustained dual‐gene vector delivery systems to treat periodontal defects. Hao et al. (2016) developed a chemical vapor deposition‐based dual‐gene delivery system, which has been demonstrated to successfully deliver Ad‐BMP‐7 and Ad‐PDGF‐B to human PDL cells, resulting in highly compartmentalized and sustained protein production compared with physical absorption. In another study, a polymeric scaffold containing Ad‐BMP‐7 with Ad‐PDGF‐B was implanted in a preclinical model of a buccal dehiscence defect, concluding that either Ad‐BMP‐7 or Ad‐PDGF‐B was capable of promoting periodontal regeneration individually. Still, their combination synergistically enabled the wound healing (Y. Zhang et al., 2015).

Although the efficiency of transfecting cells is relatively high with viral vectors, concerns surrounding immunogenicity and cytotoxicity must be acknowledged (Ramamoorth & Narvekar, 2015). Non‐viral vectors such as plasmids, which are small circular DNA structures that can replicate in the cell independently of chromosomes, have gained significant traction due to their reduced pathogenicity, low cost, and ease of production (L. Jiang et al., 2020). Although the development of gene therapy in periodontal regeneration is at the early stage and warrants additional investigation, it has demonstrated outstanding potential for catalyzing coordinated regeneration of both soft and hard tissues in the periodontium (Woo, Cho, Tarafder, & Lee, 2021).

### 5.3 Next‐generation biomaterial design for periodontal regeneration

The physical design of biomaterial scaffolds relies on physical cues to induce cell and tissue fate in regeneration, for example, texture and porosity (Swanson & Ma, 2020). Nanofibers enable increased cell adhesion to the biomaterial and an increased surface area for the adsorption of extracellular matrix proteins (R. Zhang & Ma, 2000). Porosity allows tissue integration, cell infiltration, and nutrient/waste exchange. The specific size of pores within biomaterials has been recently correlated to driving distinct tissue fates by modulating construct vascularization, extracellular matrix composition, and gene expression (Gupte et al., 2018; Swanson et al., 2022, 2021). Based on these understandings, various design motifs may be combined in spatially distinct regions to create composite scaffolds capable of regenerating tissues and their functional interfaces within complex microenvironments.

Multiphasic scaffolds may be designed to engineer the PDL and its interfaces based on architectural and biochemical composition variations throughout a construct (Jeon, Vaquette, Klein, & Hutmacher, 2014). Various examples of multiphasic scaffolds are described in Table 2. Considering the hierarchical structure of the periodontium, Ivanovski et al. summarized critical aspects of multiphasic scaffold design for periodontal tissue engineering: (a) compartmentalization of bone and PDL; (b) promotion of cementum formation on the root surface; and (c) formation of appropriately oriented PDL fibers (Ivanovski, Vaquette, Gronthos, Hutmacher, & Bartold, 2014). According to these general principles, either biphasic (bone‐PDL or PDL‐cementum) or triphasic scaffolds (bone‐PDL‐cementum) were designed previously for the integrated periodontium regeneration (Woo et al., 2021; Yao et al., 2022).

**TABLE 2:** Design and fabrication of multiphasic scaffolds for periodontal tissue engineering

CM compartment	PDL compartment	AB compartment	Main outcomes	References
Biphasic				
NA	3D‐waxing‐printed PGA scaffold	3D‐waxing‐printed PCL scaffold	Parallel‐ and obliquely‐oriented PDL fibers within the construct	Park et al. 2010 [189]
NA	Solution electrospun membrane	FDM scaffold	Cementum‐like tissue deposition at the dentin‐cell sheets interface	Vaquette et al. 2012 (Sowmya et al., 2017)
NA	Solution electrospun membrane	Melt electrospun scaffold	Tissue integration between the bone and PDL	Vaquette et al. 2019 [190]
Triphasic				
100 μm microchannel + human amelogenins	600‐μm microchannel + CTGF	300‐μm microchannel + BMP‐2	Formation of bone, PDL, and cementum/dentin‐like tissue in the various scaffold compartments with characteristic histologic features	Lee et al. 2014 (Van Steenberghe, 2000)
Chitin‐PLGA/nBGC nanocomposite + CEMP1	Chitin‐PLGA hydrogel + FGF‐2	Chitin‐PLGA/nBGC nanocomposite + PRP	Defect closure; cementum, PDL, and alveolar bone formation	Sowmya et al., 2017 (Gault et al., 2010)

Abbreviations: AB, alveolar bone: CM, cementum; PDL, periodontal ligament.

Vaquette et al. (2012) reported a biphasic tissue‐engineered construct for periodontal regeneration with a porous PDL compartment and stiff bone compartment. Excellent tissue integration between bone and PDL compartments and the tooth root interface with the establishment of Sharpey's fibers was observed. 3D‐printing techniques may also be employed in this area, as demonstrated by Park et al. (2012) in a PDL‐bone composite scaffold. An ectopic periodontal regeneration model showed a significantly more organized fibrous connective tissue with calcified tissue layers on the dentin surface in biphasic scaffolds compared to a random‐porous structure. Triphasic scaffolds have the potential for complete regeneration of the periodontium to promote the generation of two mineralized tissues and one soft tissue in the middle. In 2014, C. H. Lee et al. (2014) printed seamless scaffolds with tissue‐specific microstructures consisting of three phases, yielding aligned PDL‐like collagen fibers inserted into bone‐like tissue and putative cementum matrix protein‐positive tissues. Sowmya et al. (2017) suggested triphasic nanocomposite hydrogel scaffolds combined with three tissue‐specific growth factors (CEMP‐1 for the cementum layer, FGF‐2 for the PDL layer, and PDGF for the bone layer). The results confirmed the formation of new cementum, fibrous PDL, and alveolar bone with well‐defined bony trabeculae in a rabbit periodontal defect (Sowmya et al., 2017).

Although multiphasic scaffold strategies seem to be well‐suited for periodontal tissue engineering and regeneration in terms of their ability to stimulate coordinated responses in both soft and hard tissues, there is a scarcity of work and apparent limitations. First, as most of the multiphasic constructs were made solely from polycaprolactone, the biomaterial is hydrophobic and does not provide any specific biochemical cues. For non‐3D‐printed scaffolds, the thickness and shape of the constructs were not highly adjustable, preventing a precise match between the dimensions of the defect. As of 2022, no clinical studies have been published in the literature. Designing a multiphasic scaffold with strong cohesion between different phases, sufficient surgical handling properties, and the ability to customize the morphology of the scaffold to adapt to clinical defects of varying shapes and sizes are key points to be considered for future clinical translation (Galli et al., 2021).

In addition to the general principle of biomaterial design for periodontal regeneration, which aims for coordinated regeneration of PDL, cementum, and alveolar bone, a key question in the field of periodontal regeneration is whether a PDL tissue is advantageous for dental implants, as opposed to implant osseointegration (Giannobile, 2010). In one way, implementing hybrid biomaterials, which can promote the formation of implant‐ligament interfaces, offers tremendous potential for oral implants to maintain form, function, and proprioceptive responses more similar to a natural tooth (Van Steenberghe, 2000). On the other hand, technologies demonstrate significant unpredictability in human clinical trials and raise concerns around cost and the impractical application of cell‐based tissue engineering technologies (Gault et al., 2010).

### 5.4 The use and need for stem cells in periodontal regeneration

A key consideration in regenerative therapeutics for periodontal regeneration is the role of stem cells. Classical tissue engineering paradigms involve transplanting exogenous stem cell sources into a defect site (Langer & Vacanti, 1993). Recently, various reports have demonstrated that cell transplantation does not directly accelerate wound healing in craniofacial defects (Kitami et al., 2016). Concerning the PDL, Yan et al. (2015) showed similar regenerative results from a hydrogel loaded with PDL‐SCs and an empty hydrogel after 4 weeks of wound healing in a rat infrabony defect and concluded that the contribution of hydrogel‐incorporated cells to periodontal regeneration could not be ascertained. Chemotactic growth factors and extracellular vesicles, which recruit endogenous cells to a defect site, may be sufficient to catalyze the regeneration (Gegout et al., 2021). Chew et al. demonstrated that mesenchymal stem cell‐derived exosomes, loaded in a collagen sponge, catalyzed periodontal regeneration in a rat model by activating pro‐survival AKT and ERK signaling (Chew et al., 2019). Yue et al. similarly demonstrated that weekly exosome injections into gingival tissues suppressed pathogen‐triggered inflammatory responses by macrophages and showed their ability to be used as the most modulation agent in the management of periodontitis (Yue et al., 2022). Experiments studying the role of conditioned media and various secreted factors in the periodontium will undoubtedly lead to discoveries of bioactive molecules involved in physiologic tissue development, maintenance, and repair (Lin et al., 2021). In this way, taking advantage of endogenous cells rather than relying on the transplantation of exogenous stem cells represents a significantly decreased regulatory and cost burden for the clinical translation of these exciting new technologies. These findings highlight the importance of understanding the molecular signatures and character of the periodontal stem cell population and its neighboring stem cell populations (Pagella, de Vargas Roditi, Stadlinger, Moor, & Mitsiadis, 2021).

## 6 CHALLENGES AND FUTURE DEVELOPMENTS

Biomaterials and growth factors, and their synergies together, represent significant technologic advantages for the future of precision periodontal medicine and the predictable periodontal regeneration (Kornman et al., 2017). Growth factors can induce cell migration, tissue morphogenesis, phenotype induction, vascularization, and healing. Biomaterials provide a matrix for regeneration and facilitate the spatial and temporal controlled release of inductive substances to catalyze regeneration. An intimate understanding of the developmental biology underlying the periodontium is critical for designing and identifying promising interventional strategies which can improve patient outcomes toward complete regeneration of the periodontal structures, including cementum, PDL, and alveolar bone. This encouraging progress in these areas advances clinical translation and the use of multiple biologic therapeutics for periodontal regenerative medicine. Further development is needed to improve the predictability of these outcomes, particularly considering the long‐term stability of regenerated periodontal tissues. Periodontal regeneration has been a rapidly growing field with significant excitement since its inception, with a tremendous potential to advance periodontal healthcare.

## CONFLICT OF INTEREST

The authors declare no conflict of interest.

## Data Availability

All data related to this manuscript are provided in relevant citations and included in the manuscript.
